# Importance of surgical assembly technique on the engagement of 12/14 modular tapers

**DOI:** 10.1177/09544119211053066

**Published:** 2021-10-25

**Authors:** A Wade, F Webster, AR Beadling, MG Bryant

**Affiliations:** School of Mechanical Engineering, Institute of Functional Surfaces, University of Leeds, Leeds, UK

**Keywords:** Modular total hip replacement, taper assembly, disassembly force, seating mechanics, surgical technique

## Abstract

Fretting-corrosion at the modular taper junction in total hip replacements (THR), leading to implant failure, has been identified as a clinical concern and has received increased interest in recent years. There are many parameters thought to affect the performance of the taper junction, with the assembly process being one of the few consistently identified to have a direct impact. Despite this, the assembly process used by surgeons during THR surgery differs from a suggested ‘ideal’ process. For example, taper junctions of cutting tools should be pushed together rather than impacted, while ensuring as much concentricity as possible between the male and female taper and loading axis. This study devised six simple assembly methodologies to investigate how surgical variations affect the success of the compressive fit achieved at the taper interface compared to a controlled assembly method, designed to represent a more ‘ideal’ scenario. Key findings from this study suggest that a more successful and repeatable engagement can be achieved by quasi-statically loading the male and female taper concentrically with the loading axis. This was shown by a greater disassembly to assembly force ratio of 0.626 ± 0.07 when assembled using the more ‘ideal’ process, compared to 0.480 ± 0.05 when using a method closer to that used by a surgeon intraoperatively. Findings from this study can be used to help inform new surgical instrumentation and an improved surgical assembly method.

## Introduction

The vast majority of total hip replacements (THR) implanted today now present modularity via a tapered junction. Introduced in the 1980s, the modular taper is a ‘self-locking’ interference fit connection, that allowed the separation of the once monobloc femoral stem into two separate components, the femoral stem and femoral head. This gave surgeons the ability to mix materials of differing properties and sizes to form the optimal implant for the patient, key to a successful surgical outcome.^1–3^ With over 90,000 hip surgeries taking place in the UK every year, it is one of the most successful surgical procedures, with only 5% requiring revision after 10 years.^4^ However, the taper junction has recently received press attention and has been linked to higher than acceptable revision rates owing to mechanically assisted crevice corrosion, better known as fretting-corrosion.^5,6^

Fretting-corrosion at the modular taper interface is a complex degradation process with both mechanical (wear, fatigue) and chemical (corrosion) processes.^7–10^ This is due to the interface allowing fluid ingress and motion. Essentially, this involves the constant disruption of the passive oxide layer that spontaneously forms on the surface of metallic implants. Initially, this occurs within an oxygen-rich environment, but the taper geometry allows fluid stagnation resulting in the depletion of oxygen over time. This difference in concentration can set up a preferential anodic site within the crevice and cathodic site outside, driving the production of metal ions.^11^ The interface can become starved of oxygen and the production of metal ions can attract negatively charged anions, such as chloride ions. This can lead to the production of hydrochloric acid and cause the solution pH within the taper geometry to drop, affecting the thermodynamic stability of the passive layer and its ability to resist corrosion.^11^ Other possible degradation mechanisms that have been associated with the taper junction include stress corrosion cracking and hydrogen embrittlement.^10^ Products of degradation are commonly associated with adverse local tissue reactions, presented in patients as pain followed by instability, some studies have also suggested possible systemic implications.^12–15^ Less commonly, fretting-corrosion can also go on to cause catastrophic failure of the implant such as neck fracture or head dislocation due to excessive material loss.^1,13,16,17^

The modular taper in THR was based on a ‘self-locking’*Morse Taper*. Invented in the 1860s, the *Morse Taper* is composed of a female conical taper (spindle) and a male taper (toolholder) to allow machine parts for drills, lathes and milling machines to be changed quickly without compromising torque transmission.^18^ This is reliant on the precision manufacture of the tapered interface to ensure a uniform compressive stress distribution over the whole interface when forced together. The taper can sometimes be a weak point in the machining system as it too can allow motions at the interface for fretting-corrosion and reduced quality of the workpiece, especially when subject to high cutting forces.^18–20^ The stiffness of this junction is sensitive to the quality of the taper surfaces and the magnitude of the axial preload force.^20^ Typically, the axial forces achieved at tapered interfaces for industrial use are around 9–25 kN, with some taper interfaces that include a drawbar able to achieve 35–45 kN.^20^ Literature from the early 1900s details the extensive lengths gone to achieve a uniform distribution of stress over the whole interface, including explaining how tapers should be pressed together and not impacted with a hammer.^21^

There is a wide variation in the taper junction in THR, often differing quite significantly from those used in machining processes^18,22–24^; For example, the cone angle is often steeper in THR for a ‘self-locking’ taper junction, often presenting a relatively rough surface topography and a level of angular mismatch.^23,25^ Different designs have been found to influence performance and researchers have spent over 25 years studying what makes a ‘good’ taper interface, mostly failing to draw consistent conclusions. These factors can be summarised into three categories: the patient (i.e. biomechanics and weight), the implant (i.e. properties and materials) and assembly (i.e. surgical technique). One of the few individual factors that have been found to consistently affect taper performance was assembly.^26–36^ In summary, the studies demonstrated that increasing the assembly force of these ‘self-locking’ tapers increased: seating displacement,^26,27,32,37^ disassembly force,^26,28,29,33,35^ engagement,^36,38^ deformation at the interface,^36,38^ and in the short to medium term, a reduction in the amount of motion^27,32^ and fretting-corrosion.^27,30,31,34,37^ Other variables present during in-vivo assembly of the head and stem, such as fluid contamination, has been found to affect the taper junction. On the other hand, variables such as assembly rate have been found to have little effect.^26,30,39^ One study by Ouellette et al.^26^ conducted a detailed investigation into the seating mechanics of the taper junction. This study observed that certain sides of the head displaced further indicating that alignment changes during seating, suggesting that the tapers are ‘self-aligning’.

Despite a high sensitivity to assembly, most studies when investigating the taper junction in-vitro use a 2 kN axial force to comply with ISO 7206,^40^ often under quasi-static conditions. ISO 7206^40^ also specifies a tolerance of 0 ± 1° between the male taper axis and the loading axis, where the axis of the head taper is free to self-align. Studies that investigated the assembly forces applied by surgeons suggest a peak force of anywhere between 1 and 20 kN can be achieved, with an average of around 7 kN, much higher than 2 kN and very different to the quasi-static conditions employed by most studies.^41–43^

This study aimed to investigate how the assembly mechanics varied with different surgical techniques. This was achieved by conducting a series of simple, yet novel experiments to simulate varying assembly methods, investigating the effect of the position of the head before loading, the loading angle and the assembly rate (quasi-static vs non-quasi-static). Compressive stress achieved at the interface was measured using assembly-disassembly tests, whereby the head was assembled onto the taper and the force required to pull the head free was determined. Findings from this study can be used to help inform the development of future surgical instrumentation, improved surgical assembly methods and a more controlled assembly process for in-vitro testing for further investigation into what a ‘good’ taper junction might look like.

## Materials and methodologies

The assembly mechanics and disassembly force were investigated using a series of assembly techniques. These were devised to simulate different surgical assembly methods of the taper junction, in a controlled environment.

### Samples

**Figure 1.**
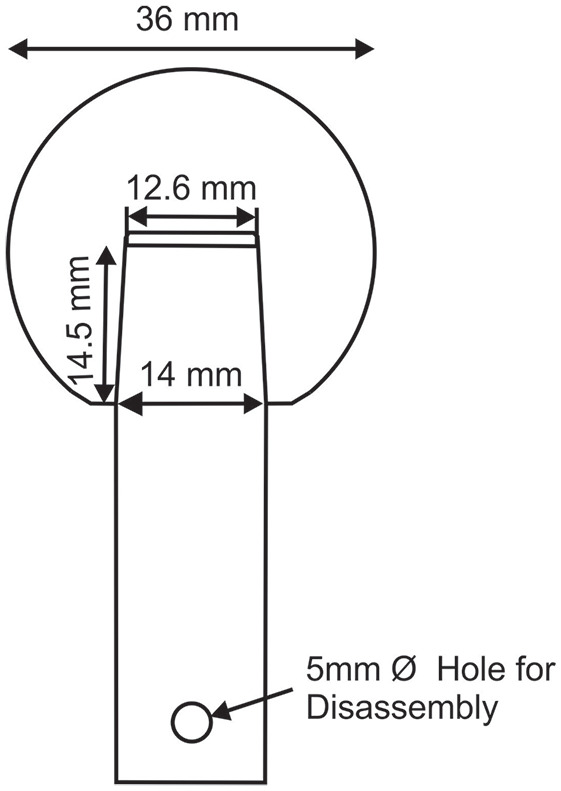
Schematic of samples with macro geometrical measurements.

The samples were all manufactured from Cobalt Chromium Molybdenum alloy (CoCrMo) and feature a clinically available 12/14 taper junction (*Aesculap*, Germany). Figure 1 shows a diagram of the components with the male taper manufactured on a simplified lower stem geometry.

**Figure 2.**
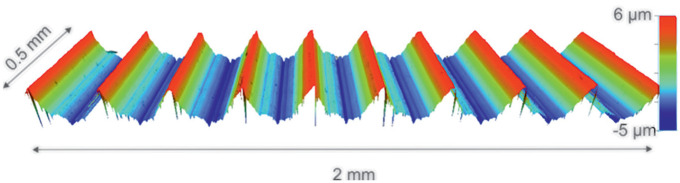
Example scan of the surface topography taken using a ×20 magnification.

Taper geometry was measured using a coordinate measurement machine (CMM) according to the protocol described by Wade et al.^23^ Surface roughness parameters describing amplitude, distribution and shape of the profile was done using Vertical Scanning interferometry (*Bruker*, USA) according to ISO 4288-98.^44^ Outputs of this analysis are shown in Table 1. The angular mismatch achieved by the couples was 0.098 ± 0.014° (range from 0.080 to 0.112°) and the surface topography presented a threaded type finish, shown in Figure 2. The taper interfaces were cleaned before each assembly with acetone and air dried to remove any debris or contaminants that could affect engagement.

**Table 1.** Taper angle and surface roughness measurements showing ± one standard deviation from all the samples used in this study. Description of each parameter can be found in ISO 4287,^45^ ISO 13565^46^ and ISO 25178.^47^

	Taper Angle (°)	S_a_ (µm)	S_z_ (µm)	S_k_ (µm)	S_sk_ (−)	S_ku_ (−)	S_pd_ (mm^−2^)
Male taper	5.668 ± 0.006	2.91 ± 0.43	13.42 ± 1.33	8.45 ± 2.82	0.26 ± 0.25	1.89 ± 0.22	3504 ± 229
Female taper	5.774 ± 0.003	–	–	–	–	–	–

### Methodology

**Figure 3.**
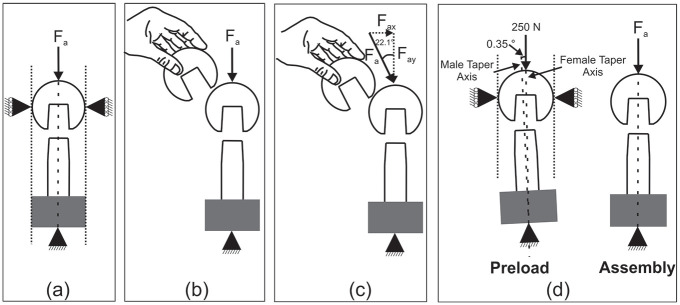
Schematics of the different assembly techniques: (a) concentric, (b) hand placed head, (c) angled loading and (d) forced misalignment. Where F_a_ represents the assembly loads.

All tests were conducted with a uniaxial material testing machine (*Instron*, US) accurate to ± 0.5% of the measured force. Four different assembly techniques were used: concentric assembly, hand placed head, angled loading and forced misalignment. The hand placed head and angled loading conditions were completed at two different assembly rates (quasi-static and high rate). Figure 3 shows the schematics of the four different techniques:

- **Concentric** assembly (Figure 3(a)), involved the use of bespoke precision manufactured fixtures to ensure concentricity between male taper, female taper and the loading axis of the material testing machine. Fixtures were manufactured to tolerances of 0.005 mm. The male taper was held concentrically with the loading axis using an appropriate boss, complementary to that on the base of the test frame. The female head was clamped using the flat surface at the opening of the taper and the pole of the spherical head, resulting in an angular tolerance of under 0.001 ° between the male and female taper with the loading axis.
- **Hand placed head** (Figure 3(b)) assembly, entailed placing the head onto the male taper before using the test frame to apply load. Placing the head and applying a force without the use of the rigs to ensure concentricity, was undertaken to study the effect of head position by the surgeon before assembly. This method was in line with ISO 7206.^40^
- **Angled loading** (Figure 3(c)) followed a similar process to the hand placed technique, except for the applied force being at an angle of 22.1° to the male and female taper axis, which were free to ‘self-align’ to simulate variations in the angle of the impaction tool to the taper axis in-vivo. It was estimated that surgical variation is around ± 10°, an angle of 22.1° was selected to avoid a false negative due to the large variation in disassembly forces shown by previous studies.
- **Forced misalignment** (Figure 3(d)) assembly comprised of a two-stage process. The first stage involved applying a preload of 250 N with an angle of 0.35° between the female and male taper axes. This was achieved using the same rig to ensure concentricity with the addition of an angle of 0.35° applied to the base plate. The second step was to remove the 0.35° angle and constraints and apply the remaining assembly load. The aim of forcing a mismatch between the taper axis with a preload was to further investigate the ability of these taper junctions to ‘self-align’ by measuring the resulting disassembly force.

The four different assembly techniques were assessed using a similar incremental push-on-pull-off method used by Ouellette et al.^26^ This study assembled samples to loads from 1 to 6 kN in increments of 1 kN. The force misalignment assembly technique was an exception and was only incremented to 4 kN due to the non-representative nature of the intra-operative assembly process. Disassembly force (F_d_) was determined at each loading increment before moving on to apply the next increment, 1 kN larger than the previous.

Two different assembly rates were used, quasi-static conditions with rate 0.04 mms^−1^ and a higher rate of assembly, reaching the peak forces (1, 2, 3, 4, 5 and 6 kN) in 0.5 s. All four different assembly techniques were assessed quasi-statically. The hand placed head and angled loading techniques were also investigated under the high rate due to them being the most representative conditions to surgical assembly. High rate was aimed at simulating an assembly more a kin to an impaction delivered during surgery. Although during surgery, the force is delivered in a much smaller time frame, around 1500 time faster.^41^ This resulted in six different assembly test conditions that were all repeated three times, with 15 different head-stem couples, one for each run. Table 2 summarises each of the six different tests.

**Table 2.** Summary of the six assembly methodologies investigated in this study.

Assembly conditions	Preload (N)	Max load/increment (kN)	Assembly rate	No. of repeats
Concentric	–	1–6/1	0.04 mms^−1^	3
Hand placed	–	1–6/1	0.04 mms^−1^	3
Quasi-static				
Hand placed	5	1–6/1	To peak force in 0.5 seconds	3
High rate				
Angled	–	1–6/1	0.04 mms^−1^	3
Quasi-static				
Angled	5	1–6/1	To peak force in 0.5 seconds	3
High rate				
Forced misalignment	250	1–4/1	0.04 mms^−1^	3

Disassembles were performed at a rate of 0.008 mms^−1^ until the tensional force registered less than 500 N. Time, force and displacement were recorded at a rate of 10 Hz for assembly and 5 Hz for disassembly at a resolution down to 0.1 mN and 0.01 µm.

### Analysis

**Figure 4.**
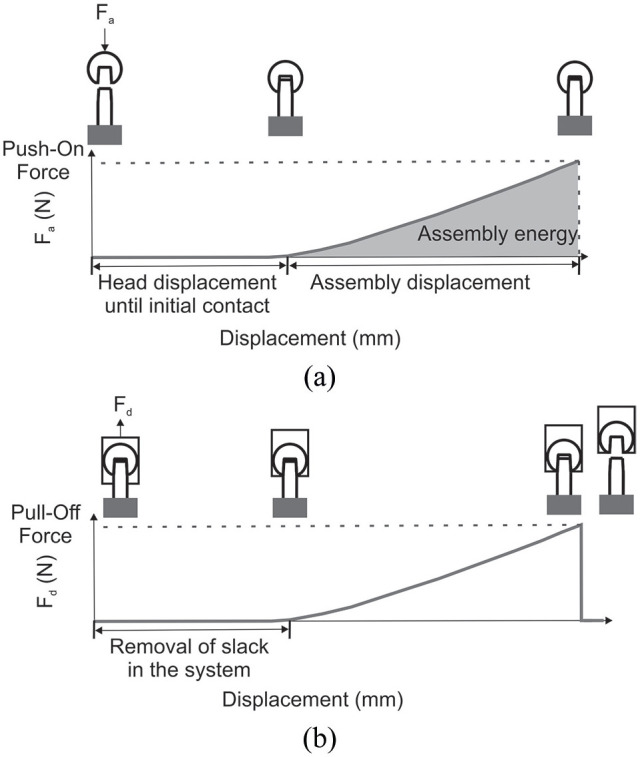
Schematic of force-displacement data from: (a) assembly (or push-on) and (b) disassembly (pull-off). Where F_a_ corresponds to the force applied during assembly and F_d_ to the force applied during disassembly.

Raw data was exported as a.*csv* file and allowed the calculation of displacement during assembly, assembly energy and pull-off force. Figure 4 shows how the different parameters were calculated from the raw data extracted from the test frame.

Assembly displacement and pull-off force (F_d_) were plotted against push-on force (F_a_) to assess how the assembly mechanics affect the success of the compressive fit. In the case of angled loading, the vertical component of the assembly force along the taper axis (F_az_) was plotted. The assembly mechanics of the forced misalignment method were not compared to the other techniques due to the assembly mechanics effectively being the independent variable and therefore not comparable to the other assembly methods. Parameters were calculated using Excel (Microsoft, US).

## Statistical analysis

Parameters were displayed as a mean of the three repeats with error bars that represent standard error (unless stated otherwise) that is, an indication of how far the calculated mean might be from the true mean based on the distribution of the three repeats. To determine the statistical difference, a one-way ANOVA, with a post hoc t-test with an alpha of 0.1 was used. Although an arbitrary critical *p*-value of 0.05 is most widely adopted throughout scientific studies, it can be said that 0.1, 0.05 and 0.01 can be considered typical.^48,49^ This study also reported actual p-values where appropriate.

## Results

### Disassembly force

**Figure 5.**
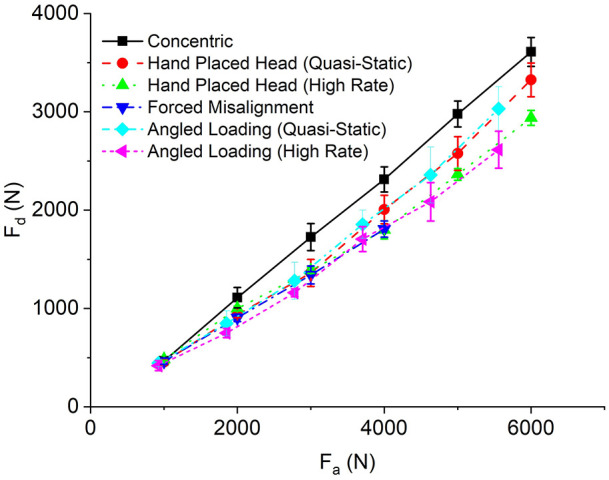
Pull-off force (F_d_) plotted against assembly force (F_a_) for each of the six assembly techniques at each loading increment. Error bar represents plus and minus standard error.

The pull-off force (disassembly force, F_d_) was plotted against the push-on force (assembly force, F_a_) and shown in Figure 5. Generally, F_d_ increased linearly with F_a_ with variations in the relationship between the different assembly methods. F_d_ was initially very similar for all assemblies but began to differ at F_a_ greater than 2000 N, shown by the gradients (i.e. Δ F_d_/ΔF_a_) in Figure 5. The concentric assembly technique resulted in greater values of F_d_, more so at higher assembly forces. The smallest values of F_d_ were recorded for the high rate hand placed head, high rate angled loading and forced misalignment assemblies. The samples assembled using the hand placed head and angled loading methodologies under quasi-static loading rates presented an improved F_d_ compared to the equivalent assemblies subject to high rate.

**Figure 6.**
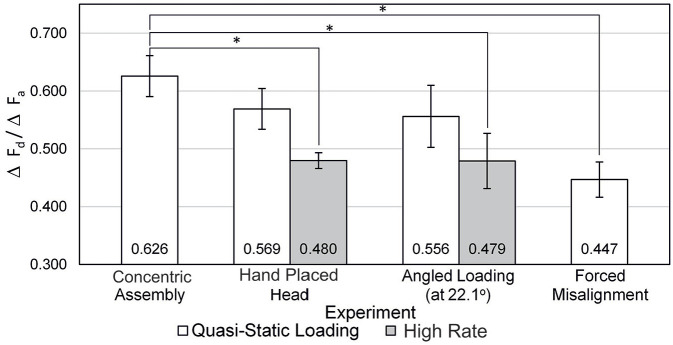
Comparison of push on pull off gradients for all experiments completed with appropriate *p*-values. Error bars represent standard error.

Figure 6 shows the average gradients from Figure 5, where the ratio of F_d_ and F_a_ was approximately between 0.458 and 0.65 depending on the assembly technique. Assemblies considered statistically different from the concentric have been indicated.

High rate loading and forced misalignment resulted in the largest decrease in the ratio of F_d_ to F_a_ out of the six assembly conditions from the concentric (*p*-value = 0.01 for hand placed head high rate, *p*-value = 0.13 for angled loading high rate and *p*-value = 0.05 for forced misalignment). Positioning the heads by hand (i.e. hand placed head and angled loading) and loading quasi-statically, only resulted in an average 9% fall in gradient (i.e. ratio of F_d_ to F_a_) from the concentric method (*p*-value = 0.28 and 0.33 for hand placed head and angled loading, respectively). Further analysis of the data shown in Figure 6 indicated that high rate assembly of the heads resulted in an approximate 14% decrease in gradient compared to loading them in the same manner quasi-statically, statistically different for the hand placed head method (*p*-value = 0.08) but not angled loading (*p*-value = 0.36). Additionally, angled loading compared to hand placed head assembly did not result in detrimental effects on F_d_, and variation between the two techniques at equivalent loading rates had a low probability of being down to assembly. However, the error bars appear to be larger in the angled loading case compared to the hand placed head, indicating higher variability with the angled loading technique.

## Assembly mechanics

The seating mechanics are believed to be intimately linked to how successful the compressive fit is at the taper junction. This section investigated the assembly mechanics of the hand placed head and angled loading methodology (quasi-static and high rate) against the more ‘ideal’ concentric method.

**Figure 7.**
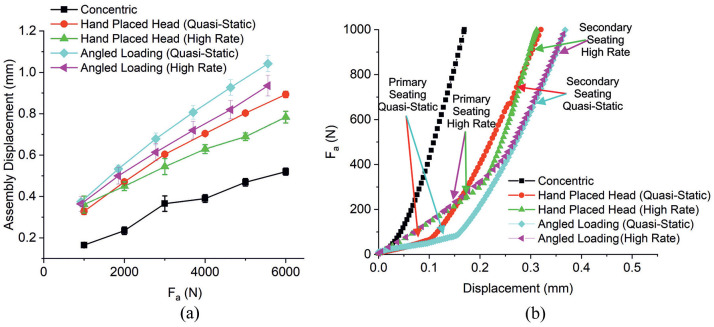
(a) Assembly displacements for concentric assembly and hand placed head and angled loaded assembles at quasi-static and high rate assembly rates with error bars that represent standard error from three repeats. (b) The initial force-displacement response of the three assembly processes shown in (a).

Figure 7(a) shows the assembly displacement with F_a_. Like F_d_, assembly displacement increased with F_a_ with an approximately linear relationship and variation was seen between the assembly methods. The concentrically assembled heads provided the smallest assembly displacements at each value of F_a_, despite providing the largest values of F_d_. This was found to be statistically different at all assembly forces, with an average *p*-value of 0.02. The other assembly methods (i.e. hand placed head and angled loading both quasi-static and high rate) all presented a similar initial assembly displacement. At greater values of F_a_, the quasi-statically loaded methods presented greater assembly displacements compared to the same method under high rate. The assembly displacements between the high rate and quasi-static loading hand placed head assembles became significantly different at F_a_ greater than 3000 N (*p*-value = 0.05, 0.03 and 0.12 at F_a_ 4000, 5000 and 6000 N, respectively). No statistical difference was observed at a given increment between angled loading quasi-static and fast rate but was found when comparing displacements at all the increments (*p*-value of 0.02). In addition, the hand placed heads presented statistically smaller assembly displacements than the angled loading assemblies at higher values of F_a_, with *p*-values of 0.05 and 0.04 at the highest assembly increment for the quasi-static and high rate respectively.

Closer inspection of the force-displacement response (Figure 7(b)) shows that the concentric assembly shows a stiffer single-phase assembly response, compared to the hand placed head and angled loading assemblies which showed a less stiff two-phased response. Hand placed head under high rate presented a transition between these two phases at a greater head displacement than that done quasi-statically (0.216 ± 0.007 mm vs 0.093 ± 0.007 mm, *p*-value = 0.01). This was accompanied by a steeper gradient of the secondary assembly phase compared to the hand placed head under quasi-static loading. Similarly, the angled loading subject to quasi-static showed a transition between the two assembly phases at 0.157 ± 0.001 mm compared to higher rate at 0.271 ± 0.032 mm (*p*-value = 0.06).

The force-displacement response for each experiment can be summarised by assembly energy. Assembly energy and displacements at an F_a_ of 6000 N for the five assembly techniques investigated in this section are summarised in Table 3. The concentric assembly resulted in the smallest assembly displacement, assembly energy and smallest variation in energy across the three repeats. The angled loading assemblies subject to quasi-static loading resulted in the largest displacement and energy, followed by the hand placed heads loaded quasi-statically, then angled loading subject to high rate and finally by the hand placed head subject to high rate.

**Table 3.** Summary of assembly displacement and assembly energy for F_a_ of 6000 N.

Loading rate	Concentric assembly	Hand placed head		Angled loading	
	Quasi-static	Quasi-static	High rate	Quasi-static	High rate
Average assembly displacement (mm)	0.520	0.893	0.784	1.042	0.935
Energy (N.mm)	1380	2060	1860	2240	1919
Standard deviation (N.mm)	±31.8	±100	±284	±147	±114

## Discussion

The success of the compressive fit achieved at the interface is believed to have an intimate link with modular taper performance in-vivo.^17,26,27,29–35^ This fit has been found to be sensitive to assembly force, and not that sensitive to other variations present in the surgical assembly technique such as assembly rate and the number of impactions. Historically, great lengths were taken to achieve a sufficient uniform compressive stress over the whole taper interface during assembly in cutting tools applications. This included pressing the taper together (preferably maintaining alignment between the male and female taper axis), not impacting them with a hammer, assembling the tapers in the same orientation each time and sufficient axial preload (sometimes achieved with the use of a drawbar in some taper designs).^21,50^ This study developed a technique of assembling the taper junctions in THR in a controlled manner which allowed the assessment of variations in surgical technique on the compressive fit.

In agreement with other assembly-disassembly studies,^26,28,33,41,42,51^ this study demonstrated that the disassembly force increased with increasing assembly force. Ouellette et al.^26^ found this relationship to vary between 45% and 63% depending on taper design (‘rough’ 9/10 and ‘smooth’ 12/14), material (CoCrMo-Ti6Al4V and CoCrMo-CoCrMo) and assembly under wet and dry conditions. Similarly, Danoff et al.^35^ found an average relationship of 45% of the assembly force. In contrast, Rehmer et al.^33^ found that for CoCrMo-CoCrMo couples, a range of 26%–68% for couples with ‘rough’ male taper topography. The key finding from this study was that this relationship varied with the different simulated surgical technique.

The concentric assembly technique was designed to create an ‘ideal’ situation in terms of maintaining alignment between the male taper, female taper and loading axis under quasi-static loading conditions. This was then used as a control in which to compare variations in surgical technique. Concentric assembly resulted in the largest forces required to free the female taper from the male taper, thought to be attributed to a more uniform distribution of compressive stress within the interface. The assembly mechanics in Figure 7(b) supports this hypothesis demonstrated by a smooth linear elastic relationship between force and displacement, as opposed to the two-phase assembly mechanics seen by hand placed head and angled loading techniques. However, the force-displacement response taken from the test frame was not a direct measure of interface stiffness and will include compliance of the whole load train. This resulted in assembly displacement providing an overestimation of seating displacements and assembly energy an overestimation of seating energy. Although, tests were performed consistently and should be comparable. A small investigation of unload data indicated a compressive stiffness of the load train to be around twice that of the assembly stiffness of the concentric assembly method, the stiffest of the assembly methods.

Despite the concentric assembly being the most controlled, the disassembly force presented a variation range (i.e. the difference in maximum and minimum value) of 450 N when assembled to 6 kN. This is in agreement with other studies that report standard deviations in the range of approximately ± 200 N.^26,29^ One possible explanation for this could be the stochastic nature of engagement at a conforming interface, meaning that engagement cannot be precisely controlled or predicted. A limitation of this study was the lack of surface topography data of the female tapers along with the use of only one type of taper. This meant that attempting to correlate surface measurements and engagement was considered beyond the scope of this study but will form part of future work.

Hand placed head and angled loading assemblies subject to a quasi-static assembly rate represent the first variant in surgical technique from the concentric control. The male taper, female taper and loading axis were not rigidly constrained like that in the concentric assembly. It was hypothesised that this would result in an inherent initial misalignment for a less uniform pressure distribution over the taper interface, compromising the compressive fit, resulting in a lower disassembly force. On average the disassembly force for the two techniques was smaller compared to the concentric assembly. There was no statistical significance (*p*-value > 0.1) between these two methods and the concentric at a given increment. Although, upon the comparison of the disassembly forces at all loading increments, both assembly techniques demonstrated a statistically lower disassembly force than the concentric technique (*p*-value = 0.0001 for the hand placed head and *p*-value = 8.4 × 10^−6^ for angled loading assembles). The angled loading assemblies demonstrated a larger assembly displacement than the hand placed head. It was thought that the off-axis vector acted to engage the taper earlier than if it was not present. These findings loosely indicate that tapers do present some capacity to ‘self-align’, albeit not to the same extent when concentrically aligned. It was also observed that there was a larger variation presented by the angled loading technique, where a standard deviation of ±386 N and range of 770 N was recorded, compared to the hand placed head with a standard deviation of ±296 N and range 549 N and the concentric method with standard deviation ±253 N and range 455 N. This suggests that these methods resulted in a less predictable taper connection than the concentric assembles, and the presence of force vectors perpendicular to the taper axis provides an additional source of variation.

Conducting the hand placed head and angled loading assemblies at high rate presents the second variant in the surgical assembly method. One point to note about the high rate assemblies was that, although the time in which the load was applied remained constant, the assembly rate increased with each increasing assembly force increment, with load rate of 2, 4, 6, 8, 10 and 12 kN s^−1^. Additionally, the assembly rates were much slower than achieved by impactions delivered intraoperatively,^41^ over 1500 times slower, but was higher than the load controlled rate specified in ISO 7206.^40^ High rate assembly of the heads had a statistically significant detrimental effect on the disassembly force at assembly forces greater than 2000 N compared to the more ‘ideal’ concentric assembly technique. This supports the working hypothesis that initial inherent misalignment could result in a less uniform pressure distribution and a reduced disassembly force. The lower disassembly force due to high rate assembly of the tapers compared to loading them in the same manner quasi-statically also suggests the possibility of a less uniform pressure distribution. Concentric and forced misalignment techniques were not assessed at high rate due to these techniques not being able to ‘self-align’ as the male and female taper axis were controlled during assembly. Besides, these techniques were not representative of the surgical assembly technique and were meant as a control or to test a hypothesis.

The assembly mechanics of the hand placed head and angled loading assemblies done quasi-statically versus high rate supported the theory that an enhanced unequal distribution of pressure could be achieved when not assembled quasi-statically. This was suggested by the transition from the first to the second phase of the two-phase force-displacement response which was achieved at a higher force and displacement for the high rate compared to the quasi-statically loaded (*p*-value = 0.01 and 0.03 for hand placed head and angled loading respectively). The first phase was believed to be attributed to the ‘self-alignment’ between the female and male taper via a slip-mechanism and some initial plastic material response from protruding asperities. The second phase was attributed to more of an elastic response as more contacting asperities engaged. The secondary force-displacement response for the high rate assembled heads was stiffer, resulting in lower assembly displacements, statistically different at assembly forces greater than 4000 N for the head placed heads (Figure 7(a) and (b)). A possible explanation for this observation is the localisation of contact stresses within the taper interface which has been designed to engage ‘proximally’, see Table 1. This angular mismatch may allow misalignment of the two taper axes whose ability to ‘self-align’ is compromised during a higher rate of assembly, exacerbated by these localised contact stresses upon engagement. In practice, this manifests itself by the test frame registering smaller displacements at a given force. The less uniform pressure distribution was believed to compromise the taper connection shown by the lower disassembly forces. One theory is that once locking of the modular taper has occurred, the ability for the taper to align is reduced.

The assembly rate affecting the assembly mechanics and disassembly force are contradictory to a paper presented by Ouellette et al.^26^ They found that assembly rate did not have a statistically significant effect on the assembly mechanics or disassembly force. This discrepancy could be for several reasons, including the use of a *p*-value threshold of 0.05 to test for statistical significance compared to 0.1 used by this study. Although *p*-value less than 0.05 is more conventional and the chance of identifying a false positive using *p*-value less than 0.1 is increased; this lower *p*-value threshold was thought to be justified by the lower probability of identifying trends in data with inherent high variability. Another contributory reason for this discrepancy was the use of a maximum assembly force increment of 4 kN as opposed to 6 kN used here. Despite studies that indicate 6 kN to be surgically relevant with the peak impaction force achieved by surgeons to range from anywhere between 1 to 20 kN.^41–43^

The forced misalignment assembly process was designed to take a controlled investigation into how ‘self-aligning’ tapers in THR are. This was achieved by forcing a misalignment to an axial load of 250 N (roughly the average force achieved by the primary assembly phase shown in Figure 7(b)), designed to create an uneven pressure distribution before releasing this controlled mismatch allowing them to ‘self-align’ for the remainder of the assembly increment. The ratio between assembly and disassembly force was found to be statistically lower than the concentric assembled and the two assemblies subject to quasi-static loading (*p*-value = 0.050 for concentric assembly, *p*-value = 0.008 for hand placed head and *p*-value = 0.052 for angled loading); but was almost indistinguishable from the two assemblies subject to high rate loading. This suggests that any ‘self-aligning’ properties ascribed to the taper junction in THR may not overcome a bad initial alignment. This finding is especially important when considering surgical assembly is likely to consist of placing the head on by hand and using a hammer to impact the heads with the axis of the force likely to be off the taper axis.

Although the relationship between assembly and disassembly force was found to be within the range seen previously, this study describes a more ‘ideal’ loading scenario compared to that used by surgeons and in-vitro studies that investigate the taper junction. One key finding was that use of this more ‘ideal’ loading technique was able to optimise the disassembly force for a given taper design. Despite the relationship between the assembly and disassembly force not being a validated parameter able to predict clinical performance, in-vitro studies suggest that greater assembly and disassembly forces can help reduce a taper junctions susceptibility to fretting corrosion.^34,37^ Early failure of the taper junction in-vivo is extremely complex and often attributed to the contribution of multiple factors, minimising as many of these possible contributions as possible can therefore be considered of clinical importance. For example, a case study by Chana et al.^52^ showed that early failure associated with a taper junction was attributed to: a mismatch in the male and female taper geometries from different manufacturers, use of an adaptor introducing more interfaces susceptible to fretting corrosion, a large diameter metal head and a stem made of TMZF with a relatively low elastic modulus, associated with allowing more motion at the interface. Therefore, although achieving an assembly method closer to the ‘ideal’ described in this study might not eliminate fretting-corrosion, it may go some way to reducing one of many possible factors that can contribute to early failure. Results from this study can also be used to help inform new surgical instrumentation and an improved surgical assembly method. Additional points that should also be considered when interpreting the outputs from this study include the greater peak forces achievable under impaction as opposed to pressing and the potential effect on the patient of altering surgical method.

A further implication of this study is the widespread used of 2 kN to assemble samples for in-vitro testing, stipulated by ASTM 1875.^53^ Firstly, studies indicate that there is a large variation in peak assembly forces achieved by surgeons, with a range of anywhere between 1 and 20 kN; and an average of the different studies falling somewhere around 7 kN.^41–43^ Secondly, many studies now use methodologies that involve peak axial forces of up to 4 kN, as informed by Bergman et al.^54^ to represent more realistic biomechanical loads. However, this study suggests that modular tapers may not have full seated with respect to the applied axial forces to simulate biomechanical loading. This could have a significant effect on studies designed to assess taper design, especially those subject to short-term investigations. Additionally, the concentric assembly methodology could provide a more repeatable sample assembly method for more controlled in-vitro testing.

## Conclusions

The purpose of this study was to investigate how different surgical assembly variables affect the engagement of the taper interface in modular THR. This was achieved by creating a controlled assembly methodology against which surgical variants could be assessed. This study found that:

- To maximise engagement, identified by an increased ratio between assembly and disassembly force, the head should be concentrically aligned with the loading axis, male taper axis and female taper axis and assembled at a quasi-static loading rate.
- Positioning the head by hand versus the controlled concentric assembly method bared no statistically significant effect on engagement when loaded quasi-statically with only a 9% reduction in the ratio of assembly force to disassembly force.
- Positioning heads by hand and loading at a non-quasi-static rate resulted in a less successful engagement, with a 20% reduction in the ratio of assembly to disassembly force compared to the concentrically assembled samples.
- Introducing an assembly force with a component vector perpendicular to the taper axis served to alter the assembly mechanics and increase the variability in the ratio of disassembly to assembly force acting along the taper axis.
